# Progress of PD-1/PD-L1 signaling in immune response to liver transplantation for hepatocellular carcinoma

**DOI:** 10.3389/fimmu.2023.1227756

**Published:** 2023-07-20

**Authors:** Feng Ju, Dawei Wang, Lan Huang, Chun Jiang, Ce Gao, Cunquan Xiong, Guanghua Zhai

**Affiliations:** ^1^ Department of Laboratory Medicine, The Yangzhou University Jianhu Clinical College, Jianhu, China; ^2^ Department of Infectious Diseases, The Second People’s Hospital of Yancheng City, Yancheng, China; ^3^ Department of Clinical Laboratory, The Affiliated Suzhou Hospital of Nanjing Medical University, Suzhou Municipal Hospital, Gusu School, Nanjing Medical University, Suzhou, Jiangsu, China; ^4^ College of Pharmacy, Jiangsu Vocational College Medicine, Yancheng, Jiangsu, China

**Keywords:** liver transplantation, HCC, PD-1/PD-L1 signaling pathway, ICI, immune tolerance

## Abstract

Primary liver cancer is one of the most common malignant tumors in China. The vast majority of primary liver cancer are hepatocellular carcinoma. Due to its high incidence and mortality from HCC, HCC has always been a feared type of cancer. Liver transplantation, as one of the important means to treat advanced liver cancer, has brought new hope to patients. However, as patients have been in a state of immunosuppression after liver transplantation, these patients face new problems of HCC recurrence and metastasis. A increasing number of studies have proved that blocking the PD-1/PD-L1 signaling pathway and restoring the immune killing inhibition of T cells can produce better therapeutic effects on tumors and chronic infectious diseases. As a promising treatment in the field of tumor immunotherapy, PD-1/PD-L1 inhibitors have achieved important results in liver cancer patients, but their application in liver transplantation patients is still highly controversial. This paper will introduce the mechanism of action of PD-1/PD-L1 signaling pathway and the current basic and clinical studies of PD-1/PD-L1 signaling pathway associated with immune response in HCC transplantation.

## Introduction

Primary liver cancer (hereinafter referred to as liver cancer) is one of the most common malignant tumors in China, and its incidence and fatality rate are among the forefront of malignant tumors in China (1–3). Liver transplantation, as one of the radical means to treat liver cancer, has brought new hope for liver cancer patients (4–6). At present, the number of liver cancer patients accounts for nearly half of the total liver transplant cases in China, much higher than that in other countries (7–12). However, the five-year survival rate of liver cancer and liver transplantation was only 46.8%, and the five-year cumulative recurrence rate was 36.7% (13, 14). Because the patients are in a state of immunosuppression after liver transplantation, once liver cancer recurrence, the tumor will grow rapidly and have multiple organ metastasis (15). The recurrence and metastasis of HCC has become the most important factor affecting the efficacy of liver transplantation (16). Several transplantation centers have used sorafenib for the treatment of liver recurrence after liver transplantation, effectively improving the tumor survival rate of patients (17). Tumor immunotherapy is one of the most promising and important achievements in the field of cancer therapy in the 21st century (18). As a star drug in the field, programmed. death receptor inhibitors provides a new treatment option for patients with new treatment options (19).

A growing number of studies have demonstrated that the expression of programmed cell death receptor 1 (PD-1) and its ligand (PD-L1), a new target for tumor immunotherapy, can reflect the objective response rate (ORR) and overall survival (OS) of some cancer patients. Within the tumor body, specific antigens produced by tumor cells are targeted to activate antigen-specific effector T cells (20). Then activated T lymphocytes express PD-1 on their surface and produce interferons to induce expression of PD-L1 in various tissues. Activation of the PD-1/PD-L1 signaling pathway induced apoptosis of antigen-specific effector T cells (21). Therefore, the inhibition of PD-1/PD-L1 signaling pathway can be used as an important way for immunotherapy of tumor and chronic infectious diseases (22). The signaling pathway participates in the immune escape mechanism of tumor and virus (23). Such being the case, we can use anti-PD-1/PD-L1 monoclonal antibody to block PD-1/PD-L1 signaling pathway to restore the immune killing suppression function of T cells (24). The way can produce good treatment effect on tumor and chronic infectious diseases. We have to admit that it has a good application prospect.

In recent years, immune checkpoint inhibitors (ICI) represented by PD-1/PD-L1 inhibitors have become one of the important means to treat patients with advanced liver cancer, but it is still controversial whether they can be used for liver transplant recipients. Next, we focused on the current status and trend of basic and clinical research related to PD-1/PD-L1 signaling pathway and immune response to liver transplantation for liver cancer.

## The PD-1/PD-L1 signaling pathway and its biological functions

**Figure 1:**
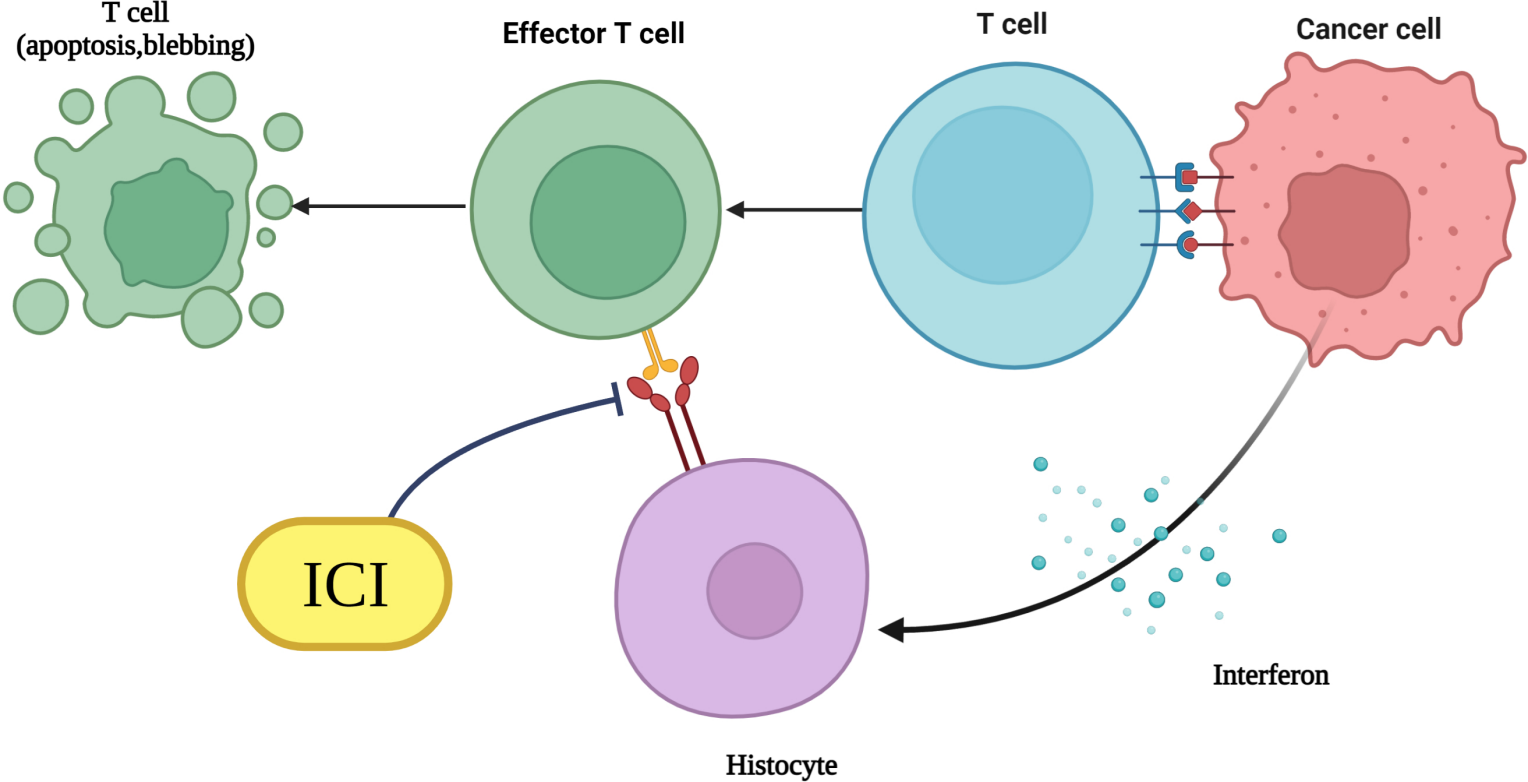
Mechanism diagram of immunotherapy with PD-1/PD-L1 monoclonal antibodies.

Programmed cell death receptors is a kind of 50~55 kD type I transmembrane glycoprotein, belonging to the member of the immunoglobulin superfamily (25). Its remarkable characteristic is that the cytoplasmic region contains two tyrosine residues, N-terminal and C-terminal (26). The former involved in forming an immune receptor tyrosine inhibition motif, while the latter is involved in forming an immune receptor tyrosine conversion motif, in which ITSM plays a key role in the negative regulation of PD-1 (27). PD-1 exists on the cell surface as monomers, and is first expressed in double-negative cells in the thymus, but also in activated T cells, B cells, natural killer cells, dendritic cells, and activated monocytes (28). PD-1 usually forms the signaling pathway with its ligands, and its ligands are PD-1 and PD-2, which share 40% amino acid sequence identity and IgC and IgV type domains in the extracellular region (29). They have similar structures but different distributions.PD-1 is widely distributed and expressed in bone marrow cells cultured from murine T cells, B cells, dendritic cells, macrophages, and mesenchymal stem cells (30). Although the nucleotide sequences of human and mouse PD-1 have 70% identity, both encode a protein of 288 amino acid residues, and have 60% identity at the amino acid level, the expression of human PD-1 is lower than that of murine source (31). The PD-2 distribution is relatively limited and is mainly expressed in activated monocyte-macrophages and dendritic cells (32). PD-1 has negative immunomodulatory effects with its ligands (mainly PD-1). PD-1 is a negative regulator of the immune response *in vivo*. When it is combined with its ligand, the tyrosine in the ITSM region is phosphorylated, and the protein tyrosine phosphatase molecules are recruited to dephosphorylate the downstream effector molecules and transduce negative signals (33). Thus, it can play a negative regulatory role (**Figure 1**).

## Expression of PD-1/PD-L1 in liver transplantation tissue

In a mouse liver transplantation immune tolerance model, C57BL/6 mice liver was transplanted into C3H mice, and more than 85% of the mice survived over 100 d (34). With C57BL/6 mice knockout of PD-L1, the liver of mice was transplanted into C3H mice, rejection occurred within 7 d, a large number of infiltrating T cells appeared in the transplanted liver, and the apoptosis level of CD8 + T cells and CD4 + T cells was low (35). PD-L1 positively associated with immunosuppressive macrophages and macrophage-derived cytokines, which may contribute to the polarization of macrophages. Moreover, inflammatory response activity exhibited significant differences between high and low PD-L1 expression groups and had robust positive correlativity of the infiltration level of tumor-associated macrophages (36). Liver allograft recipient mice in the PD-1 inhibitor-treated group developed rejection within 29 d, suggesting that inhibition of the PD-1/PD-L1 pathway would enhance the recipient immune response to the donor, leading to rejection (37). Recipient mice treated with PD-L1 inhibitor after liver transplantation showed a high inflammatory response, with phlebitis, biliary intraepithelial lymphocyte infiltration, hepatic congestion, and hemorrhagic injury after 8 d after surgery (38). Compared with 5 d after surgery, CD8 + T cell infiltration increased in the transplanted liver tissue at 8 d, and the expression of related genes (e. g., granzyme B, FasL, perforin) and inflammatory factors (e.g. osteopontin, inducible nitric oxide synthase) increased (39).

Clinically, the expression of PD-L1 is upregulated after donor liver reperfusion, especially in the portal region (40). PD-L1 was low-expressed or not expressed in donor liver biopsy tissue (biopsy) before liver transplantation (41). PD-L1 was significantly increased in bile duct epithelial cells, infiltrating T cells and hepatic sinusoid vascular endothelial cells, but the expression level in PD-L1 was not significantly changed in liver cells (42). Compared with immunotolerant recipients after liver transplantation, the number of CD4+T cells and CD8+T cells in peripheral blood of recipients with acute rejection (AR) is obviously decreased and numerous CD4 + T cells and CD8 + T cells infiltrate the liver along with decreased CD152 and PD-1 expression on CD8 + T cells and CD4 + T cells (43). The recipients of AR also showed significantly increased granzyme B and perforin expression on their CD152 and PD-1 positive CD8 + T cells (44). A biopsy of the transplanted liver from AR recipients found increased expression of PD-L1 in the portal vein and lobular areas and PD-L1 was expressed in bile duct epithelial cells in the portal region, infiltrating T cells, and endothelial cells in the lobular area, respectively (45). PD-L1 from bile duct epithelial cells, infiltrating T cells, and hepatic sinusoidal endothelial cells were present on the cell surface and in the cytoplasm, whereas PD-L1 from hepatocytes was present only in the cytoplasm (42). Current clinical reports show that PD-1/PD-L1 expression is up-regulated in liver tissue when liver transplant recipients develop AR, which may be a negative feedback protective mechanism for the body (46).

Ueki et al. simulated ischemia-reperfusion injury in liver transplantation with 24 h cold storage to constructed the mouse liver transplantation model and found that PD-L1 was expressed on dendritic cells and sinusoidal endothelial cells in the liver tissue before liver transplantation in wild-type mice, but not on liver cells, while PD-L1 was up-regulated on the above three cells in liver transplanted of recipient mice (47). In contrast to liver transplantation between wild-type mice, after transplanting the liver of PD-L1 knockout mice into the wild-type, the liver transplanted showed infiltration by lymphocytes dominated by CD8 + T cells (48). With infiltration, liver injury was significantly aggravated, the proportion of CD8 + T cells in the blood of recipient mice increased significantly, and the level of inflammatory factors such as interleukin (interleukin, IL) -6 increased significantly (49). This suggests that the PD-1/PD-L1 pathway plays an important role in reducing the postoperative immune response and protecting the liver from immune damage in the mouse liver transplantation immune response.

## PD-1/PD-L1 and transplantation immune tolerance

PD-1/PD-L1 plays an important role in inducing graft immune tolerance and promoting graft survival. During the formation of transplantation immune tolerance, T cells play an irreplaceable role, and its activation requires the co-stimulation of the first and second signals (50). The second signal, the “co-stimulatory signal”, includes not only the signals that provide positive immunity (CD28-H7, CD40-CD154), but also many immunosuppressive signals (CTLA-4, PD-1/PD-L1, OPG, DcR 3, BTLA/B7x) (51). If the positive stimulation signal provided by positive molecules is dominant, T cells will activate proliferation and differentiate into auxiliary T cells to improve immune activity (52). Conversely, immune tolerance is gradually formed.

**Figure 2:**
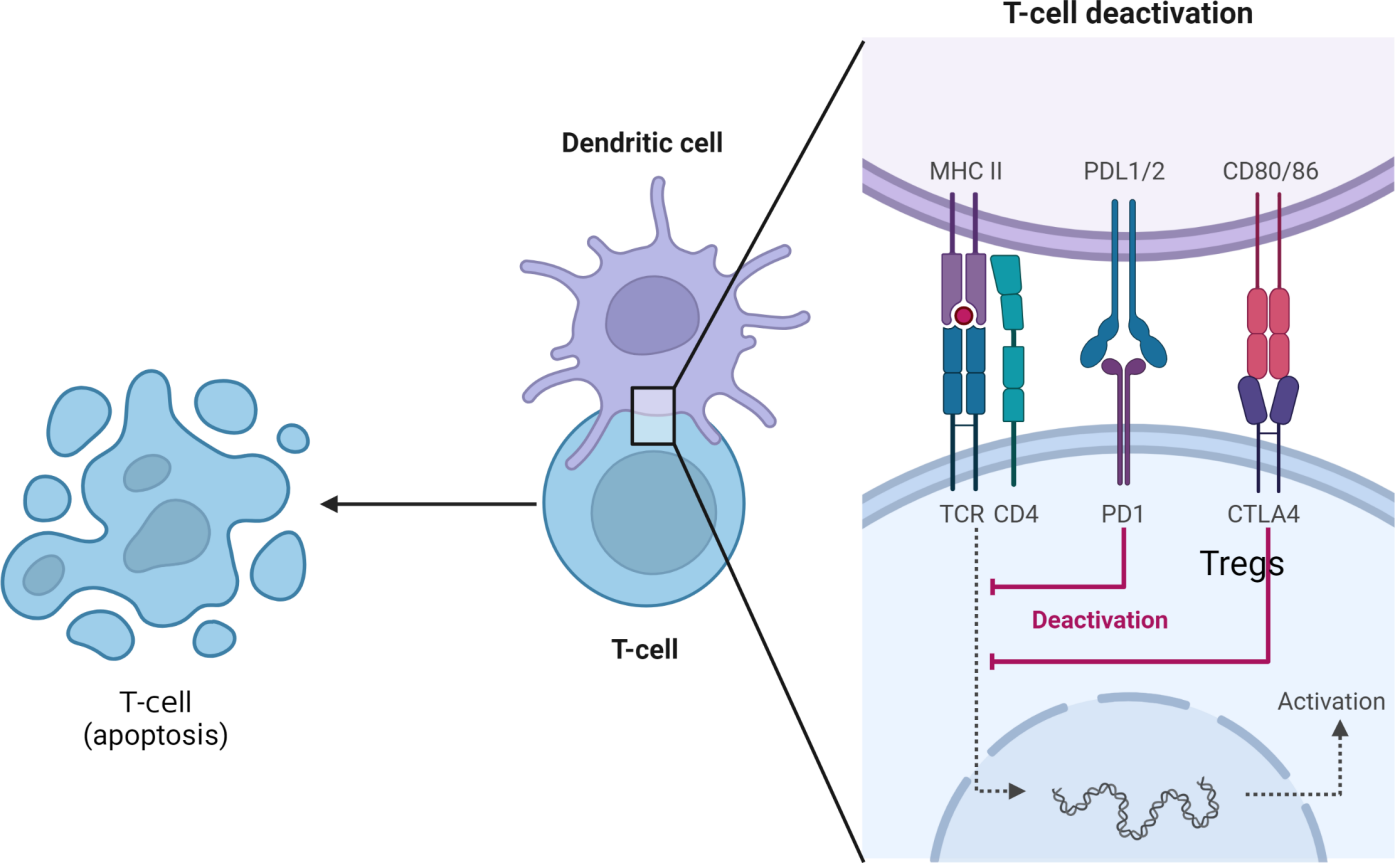
Inhibiting effector T cells and inducing their apoptosis and Tregs generation.

The PD-1/PD-L1 signaling pathway negatively regulates T lymphocytes of the immune system, and plays an important role in inducing effector T cell differentiation towards Tregs, inhibiting T cell activation and inducing apoptotic T cell apoptosis (**Figure 2**). In a mouse liver transplantation model, high expression of PD-L1 molecules by donor cells was closely associated with apoptosis of infiltrating T cells in the transplanted liver, while blocking PD-L1 signaling pathway or knocking down donor PD-L1 caused significant lymphocyte infiltration in the graft, accompanied by hemorrhage and necrosis, and death of recipient mice within 12 days (53). Among 35 liver transplant patients, the surface expression level of CD8 + T cells and CD4 + T cells in 20 patients without acute rejection was significantly higher than that of 15 patients with acute rejection, suggesting that PD-1 molecules play an important role in maintaining immune tolerance of the transplanted liver (54). Shi et al. study showed that, on the one hand, graft rejection can activate T lymphocytes, NK cells, B lymphocytes and monocytes surface PD-1 expression upregulated, on the other hand, the application of immunosuppressive agents can also induce the increase ofPD-1 expression level, indicating that PD-1 plays an important role in transplant immune tolerance (40). Its mechanism may be that on the one hand, the inhibitory signal is transmitted to produce inhibitory effect, on the other hand, the it makes Tregs play a role. Compared with CTLA-4, another negative costimulatory molecule, CTLA-4 acts in the induction period of immune tolerance, while PD-1 acts in the maintenance phase of immune tolerance; CT-LA-4 suppresses the production of high adhesion T cells, while PD-1 suppresses the immune response of T, and B; CTLA-4 and PD-1 play both coordinated and distinct roles in the formation of immune tolerance (55).

Alternatively, NK cells are innate immune cells and PD-1 is also expressed on NK cells. Subrahmanyam et al. found that functional subsets of NK cells were more active in samples responding to PD-1 inhibitor treatment than nonresponders, suggesting that PD-1 inhibitors may enhance the tumor killing effect of NK cells (56). Memory T cells have existed *in vivo* for a relatively long time. After activation stimulated by specific antigens, it can be rapidly transformed into effector T cells, which can participate in cellular immunity and play anti-tumor roles (57). Edwards et al. found that after tumor patients were treated with PD-1 inhibitors proliferation of Memory T-cell which can express CD69 and CD103 and increased numbers of CD8 + T-cells in tumor tissue were associated with improved patient survival (58). DCs are important in presenting specific antigens and activating initial T cells. It was shown that a large number of dendritic cells expressing the primary histocompatibility complex (major histocompatibility complex, MHC) I after liver transplantation replaced the DCs expressing donor MHC I, which highly expressed PD-L1 (59). Transplanted liver DCs showed reduced ability to stimulate initial T cells, significantly inhibited the recipient T cell response and promoted CD8 + T cell exhaustion or necrosis, thus inducing graft immune tolerance (60). In the mouse liver graft rejection model, the number of DCs was less in the rejection group than without rejection and PD-L1 expression on DCs was significantly reduced.

## Application of PD-1/PD-L1 inhibitors in liver transplant recipients

In recent years, ICI has brought new hope for patients with malignant tumors. But for organ transplant recipients, there is a risk of causing AR, which limits the application of this class of drugs in transplant recipients. Common ICIs include the PD-1 inhibitors pabolizumab and navumab, the PD-L1 inhibitor atilizumab and dvizumab. When ICI is applied in recurrent recipients after liver transplantation, the amount of immunosuppressive agent is usually reduced by half in order to enhance the anti-tumor effect, which further increases the risk of rejection (61). It was found that 45% (13/29) of transplant recipients receiving ICI developed AR, and 37% of 11 liver transplant recipients developed AR (62). The adverse effects of ICI use after liver transplantation can be abdominal pain, high fever, jaundice, diarrhea, and abnormalities of various liver enzymes (63). Graft loss is a very serious complication in recipients using ICI, with irreversible fulminant liver failure during the medication, resulting in graft failure (64). However, some studies have also reported that liver transplant recipients have achieved good efficacy after the application of PD-1 inhibitors, but also without developing AR (62).

Varkaris et al. reported a liver transplant recipient treated with pabolizumab for recurrent liver cancer with no significant changes in liver function, no AR after treatment, and finally died of tumor progression 9 years after surgery (65). The authors believe that for recipients of PD-1 inhibitors, the combination of corticosteroids and rapamycin can prevent AR (62). In the above two reports, the recipients who relapsed after liver transplantation used PD-1 inhibitors and achieved prolonged survival, and did not develop AR (66). The study of Nordness et al. reported that after nearly 2 years of navumab treatment, AFP decreased from 25,500 μ g/L to 5.5 μg/L, and imaging indicated significant tumor shrinkage and met Milan criteria (67). Subsequently, liver transplantation was performed, but AR appeared 6 d after surgery and died 10 d after surgery. Rammohan et al. reported that living transplanted patients with liver cancer developed lung metastasis after surgery, the patient take a turn for the better after 1 year of sorafenib treatment, and received pembrolizumab under the condition of immunosuppressant (rapamycin combined with small dose tacrolimus), with remarkable effect and imaging tumor-free survival for 10 months (68). Munker et al. summarized the published outcomes of 14 liver transplant recipients receiving CPI, 4 with graft rejection, 3 with fatal rejection within 3 weeks after the initial dose of CPI (69). Of the 12 cases with evaluable survival, the median survival time was 1.2 months. But among patients who showed a significant anti-tumor response, the survival time was 4 months versus 18 months (**Table 1**).

**Table 1:** Clinical trial of PD-1/PD-L1.

Authors	Patient	Therapeutic schedule	Results	Conclusions
Varkaris et al	Liver transplant recipients	Pembrolizumab(PD-1 inhibitor)	No significant change in liver function; No AR; Died of tumor progression 9 years after surgery;	For recipients using PD-1 inhibitors, the combination of glucocorticoids and rapamycin prevents AR
Nordness et al	With recurrent liver cancer after hepatectomy	Opdivo for nearly 2 years	The AFP was decreased from 2 500 μg/L to 5.5 μg/L; A significant reduction in tumor size; Liver transplantation was followed, but AR developed 6 d and died 10 d.	
Rammohan et al	Living liver cancer transplant patients Postoperative lung metastasis	1 year of sorafenib treatment; Pabolizumab was administered in the presence of an immunosuppressant (rapamycin combined with small dose tacrolimus)	Radimaging tumor-free survival for 10 months	
Munker et al	14 liver transplant recipients	CPI	4 cases had graft rejection, 3 of which were fatal rejection, and the rejection occurred within 3 weeks of the initial administration of the CPI; Of the 12 cases with evaluable survival, the median survival time was 1.2 months; In patients with a significant anti-tumor response, survival was 4 months versus 18 months.	

In addition, combinations of ICI with mTOR inhibitors, BARF/mitogen-activated protein kinase (MEK) inhibitors, and Bruton’s tyrosine kinase (BTK) inhibitors are also under study. When treated with PD-1 inhibitors, changing tacrolimus to mTOR inhibitors such as rapamycin and combining it with low-dose corticosteroids can reduce the risk of rejection. For AR arising after applying ICI treatment, early studies reported that 70% to 80% can be relieved by high-dose glucocorticoids, but sufficient evidence is lacking (70). For acute humoral rejection after liver transplantation, we can remove ICI from the circulation by means of plasmapheresis, which may potentially help for AR caused by ICI therapy (62). Overall, effective treatments for AR occurring after ICI treatment remain to be studied. In terms of AR prophylaxis, the available evidence suggests that the longer the interval between liver transplantation and ICI treatment, the lower the incidence of AR, which can be prolonged by the rational use of ICI and immunosuppressive agents (61).

## Summary and outlook

PD-1/PD-L1 tumor immunotherapy is one of the most popular and most promising treatments in the field of cancer therapy, which has achieved remarkable results in prolonging patient survival and improving patient prognosis. PD-1/PD-L1 is involved in immune tolerance in liver transplantation through several mechanisms. PD-1/PD-L1 inhibitors have become one of the treatments for advanced liver cancer. But for recipients with recurrence after liver transplantation, although PD-1/PD-L1 inhibitors can prolong the survival of recipients, have the risk of inducing fatal AR. When liver transplant recipients decide to undergo CPI immunotherapy, they must weigh graft loss, fatal organ failure and possible tumor response rates caused by acute rejection, choose carefully and start as soon as possible. A deeper understanding of the factors and mechanisms leading to the occurrence of rejection has important clinical implications for guiding liver transplant recipients to receive CPI therapy. To date, the specific mechanism of action of PD-1/PD-L1 pathway in immune tolerance of liver transplantation remains to be studied, and how to improve the safety of ICI medication in recipients after liver transplantation. From the perspective of the mechanism and clinical application of PD-1/PD-L1 signaling pathway in liver transplantation, the prospect of regulating PD-1/PD-L1 signaling pathway for immunotherapy is attractive.

## Author contributions

FJ, DW and LH had the idea and drafted the work. CJ and CG performed the literature search. CX and GZ critically revised the work. All authors contributed to the article and approved the submitted version.
